# Dietary Factors and Development of Impaired Glucose Tolerance and Diabetes in a General Japanese Population: The Hisayama Study

**DOI:** 10.2188/jea.13.251

**Published:** 2007-11-30

**Authors:** Yutaka Kiyohara, Akiko Shinohara, Isao Kato, Tomoko Shirota, Michiaki Kubo, Yumihiro Tanizaki, Masatoshi Fujishima, Mitsuo Iida

**Affiliations:** 1 Department of Medicine and Clinical Science, Graduate School of Medical Sciences, Kyushu University.; 2 Department of Health Promotion, School of Health and Nutrition Sciences, Nakamura-Gakuen University.

**Keywords:** alcohol drinking, fatty acids, food habits, glucose intolerance, prospective studies

## Abstract

BACKGROUND: There have been few prospective studies on diet and glucose abnormalities as determined by oral glucose tolerance test.

METHODS: To investigate the impact of dietary factors on the development of glucose intolerance including diabetes and impaired glucose tolerance, we performed a follow-up survey of 1,075 subjects aged 40-74 years of normal glucose tolerance from 1988 through 1993/1994 by repeated 75 g oral glucose tolerance test and dietary survey. Information on habitual food consumption was obtained using a semiquantitative food frequency method.

RESULTS: Of the total subjects studied, 119 (11.1 %) developed impaired glucose tolerance and 24 (2.2 %) developed diabetes during the follow-up. At baseline, the age-adjusted amount of alcohol intake was significantly higher in males who developed glucose intolerance than in those who did not (26.7 g vs. 15.7 g, p<0.05), while the polyunsaturated/saturated fatty acids (P/S) ratio was significantly higher in females with future glucose intolerance (1.42 vs. 1.31, p<0.05). Among the female subjects who developed glucose intolerance, the intake of animal fat less decreased during the follow-up period compared with normal subjects, resulting in a significant decrease in the P/S ratio (-0.09 vs. 0.05, p<0.05). In a multiple logistic regression analysis, alcohol intake at baseline for males and decreased P/S ratio during the follow-up for females remained a significant risk factor for glucose intolerance independent of other dietary and non-dietary factors as well.

CONCLUSIONS: These results suggest that a high intake of alcohol and a decreased P/S ratio contribute to the risk of glucose intolerance in contemporary Japanese.

Diabetes mellitus appears to confer an excess risk of cardiovascular disease and premature death. In Japan, the prevalence of diabetes has increased over the past several decades.^1^^,^^2^ Although Japanese are thought to be predisposed to diabetes,^3^^,^^4^^,^^5^ it is conceivable that the recent increase in diabetes in Japan is due largely to westernization of the Japanese life-style.^2^ Among the life-style factors that might influence the development of diabetes, dietary intake is one of the most important. In the literature, a number of studies have presented cross-sectional associations of dietary intake with glucose levels^6^^,^^7^^,^^8^^,^^9^ and hyperinsulinemia,^6^^,^^10^^,^^11^^,^^12^ but there have been very few studies of Japanese. Moreover, only a few prospective studies on diet and glucose abnormalities have been reported,^13^^,^^14^^,^^15^ and those using an oral glucose tolerance test (OGTT) have been fewer still even in western countries.^16^^,^^17^

In 1988, we performed a prevalence study of diabetes and impaired glucose tolerance (IGT) using OGTT in a defined general Japanese population, Hisayama.^18^ The present follow-up study used OGTT and nutritional surveys to investigate the longitudinal association between dietary factors and the development of diabetes and IGT among subjects having normal glucose tolerance at baseline.

## METHODS

### Study population

The town of Hisayama is a suburban community adjoining the city of Fukuoka, a metropolitan area on Kyushu Island in Japan. The population of the town is approximately 7,500 and has scarcely changed over the last 40 years. According to the census information for this period, the population of the town, in terms of age, sex, and occupational distribution, reflects the population of Japan as a whole.^18^^,^^19^ Full community surveys of the residents aged 40+ years were repeated since 1961.

The diabetes prevalence study was performed by simultaneously using a 75 g OGTT and a nutritional survey in a screening examination from June 29th through November 14th in 1988. A detailed description of this survey was published previously.^18^ Briefly, among the total of 3,227 Hisayama residents aged 40 to 79 years, 2,587 (80.2%) took part in the examination. After excluding 82 non-fasting subjects, 10 subjects under insulin therapy, and 15 subjects who could not complete the OGTT due to complaints of nausea or general fatigue during ingestion of a glucose solution, 2,480 remaining subjects (1,073 males and 1,407 females) completed the OGTT and the nutritional survey. In accordance with the 1998 World Health Organization criteria,^20^ these participants were classified into three groups: a normal glucose tolerance, an IGT, and a diabetes mellitus group. The criteria are as follows: for diabetes, fasting plasma glucose 7.0+ mmol/L or 2 hr plasma glucose 11.1+ mmol/L in the OGTT; for IGT, fasting plasma glucose <7.0 and 2 hr plasma glucose 7.8-11.0 mmol/L; for normal glucose tolerance, fasting glucose <7.0 mmol/L and 2 hr glucose <7.8 mmol/L.

### Follow-up examination

Among the 1,625 persons (672 males, 953 females) aged 40 to 74 years found to have normal glucose tolerance in 1988, 1,075 (66.2 %; 410 males, 665 females) also participated in the follow-up examination performed in 1993/1994, in which we repeated the OGTT and nutritional survey in the same manner as in the initial screening (Figure). Among these subjects, 119 (11.1 %) developed IGT and 24 (2.2 %) developed diabetes during this follow-up period, while the remaining 932 subjects remained normal. In the present study, we compared dietary factors at entry and their subsequent changes between the subjects who had developed glucose intolerance (GI, which means diabetes or IGT) by the time of the 1993/1994 survey and those who had not.

**Figure.**
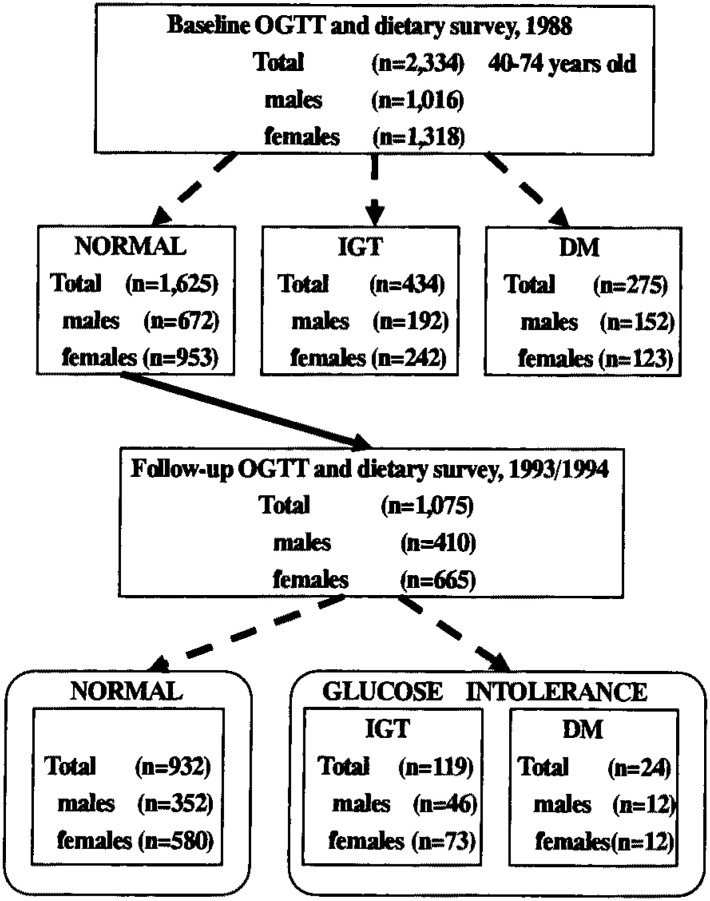
Flow diagram of the study. OGTT: oral glucose tolerance test IGT: impaired glucose tolerance DM: diabetes mellitus

### Nutritional survey

The dietary survey was conducted using a semiquantitative food frequency method,^21^ and the nutritional elements were adjusted for energy intake using the method of Willet and Stampfer.^22^ A self-administered questionnaire concerning food intake over the previous year, which consisted of 70 food items, was completed prior to the start of the study by each participant and was checked by experienced dieticians and nutritionists by showing food models of actual size in the survey. The average food intake per day was estimated based on detailed descriptions of the frequency of eating and the quantity of each food. Nutritional intake was calculated using the fourth revision of the Standard Tables of Food Composition in Japan^23^ and its follow-up version for fatty acids, cholesterol, and vitamin E.^24^ In regard to foods which were not included in these Tables, we estimated the amounts of nutritional elements by using the similar foods included in the Tables.

### Laboratory testing and risk factor measurement

Blood samples were collected from an antecubital vein after an overnight fast for the determination of the serum insulin, serum lipid, and plasma glucose levels. After the fasting blood specimen was taken, each subject ingested a 75 g glucose-equivalent carbohydrate load (Trelan G, Shimizu Pharmaceutical, Shimizu, Japan) from 8:00 a.m. through 10:30 a.m. At 120 min after glucose loading, second blood sample was obtained to determine the postloading plasma glucose. Subjects receiving oral hypoglycemic agents were requested to avoid taking their medication until completion of the OGTT. The blood specimens were transferred immediately in ice-cooled containers to the central study laboratory (Japan Medical Laboratory Inc., Fukuoka, Japan) and were analyzed within 24 hours. The plasma glucose level was measured by the glucose-oxydase method using a glucose autoanalyser (Glucoroder-MK2; A & T Inc., Tokyo, Japan). The serum insulin level was determined by a commercial double-antibody solid-phase radioimmunoassay (Phadeseph Insulin; Pharmacia Diagnostics AB, Uppsala, Sweden). The total cholesterol, HDL-cholesterol, and triglycerides were all measured enzymatically using an autoanalyser (TBA-80S; Toshiba Inc., Tokyo, Japan).

Body height and weight were measured with subjects wearing light clothing without shoes, and the body mass index (kg/m^2^) was calculated. A questionnaire concerning physical activity during leisure time and smoking habits were also administered for all subjects. Those subjects engaging in sports at least once a week during their leisure time made up physically active group. Finally, subjects were classified into two groups according to their smoking habits: a habitual use and a non-habitual use group.

### Statistical analysis

The SAS^®^ statistical software package was used for data analysis. Because of the skewed distribution of serum triglycerides and insulin, these variables were log-transformed for statistical analysis. Mean values were compared using the least significant difference analysis after adjustment for age by the covariance method. The age-adjusted frequencies were calculated using the direct method and were compared by the Mantel-Haenzel chi-square test. We estimated the multivariate odds ratio and 95% confidence interval of each risk factor, including that of dietary nutrients, using beta coefficients from the stepwise multiple logistic regression model, with p<0.1 being required for entry into the model and for remaining there.

This study was conducted with approval of the ethical committee of Kyushu University.

## RESULTS

### Characteristics of the subjects

Table 1 shows age-adjusted mean values or frequencies of baseline non-dietary factors in the subjects who did and did not develop GI by sex. The female subjects who developed GI were significantly older than those with normal glucose tolerance (58 vs. 54 years, p<0.01), but were not for the male subjects. The GI subjects had a higher body mass index and systolic and diastolic blood pressures compared with normal subjects in both sexes; the differences in these factors, with the exception of female diastolic blood pressure, were statistically significant (p<0.05). Both male and female GI subjects had significantly higher fasting plasma glucose levels than their corresponding normal subjects (p<0.01), and serum fasting insulin levels were significantly higher in male GI than normal subjects (p<0.01). The mean values of triglycerides were also significantly higher in the GI group than in the normal group for males (p<0.01), while no significant difference was found among the groups for total and HDL cholesterols in both sexes. No significant difference in leisure-time physical activity was found between GI and normal subjects of either sex. Male smokers were more frequent than female ones; however, there was no difference between the glucose tolerance groups in either sex.

**Table 1.** Age-adjusted baseline characteristics of study subjects by sex and glucose tolerance status.

Variable	Males						Females					
	
	Normal			Glucose Intolerance			Normal			Glucose Intolerance		
	n=352			n=58			n=580			n=85		
Age(years)	55	±	0.5	56	±	0.9	54	±	0.3	58	±	0.9**
Height(cm)	162	±	0.3	162	±	0.7	150	±	0.2	150	±	0.5
Weight(kg)	59	±	0.4	62	±	1.0*	51	±	0.3	53	±	0.7
Body mass index(kg/m^2^)	22.5	±	0.1	23.6	±	0.3**	22.8	±	0.1	23.5	±	0.3*
Systolic blood pressure(mmHg)	128	±	0.9	136	±	2.2**	124	±	0.7	133	±	1.8**
Diatolic blood pressure(mmHg)	78	±	0.5	83	±	1.3**	74	±	0.4	76	±	1.1
Fasting glucose(mmol/l)	5.4	±	0.02	5.8	±	0.06**	5.3	±	0.02	5.6	±	0.05**
Fasting insulin(pmol/l)	30	±	0.9	40	±	2.0**	35	±	0.7	40	±	1.9
Triglycerides(mmol/1)	1.36	±	0.05	1.90	±	0.14**	1.09	±	0.03	1.24	±	0.08
Total cholesterol(mmol/l)	5.10	±	0.05	4.90	±	0.13	5.50	±	0.04	5.50	±	0.11
HDL cholesterol(mmol/l)	1.25	±	0.02	1.20	±	0.04	1.35	±	0.01	1.35	±	0.03
Physically active on leisure time(%)	25			16			14			19		
Smoking(%)	47			52			4			6		

Means ± SEM or frequencies

Glucose Intolerance: impaired glucose tolerance and diabetes by the 1998 World Health Organization criteria

**: p<0.01, *: p<0.05 vs Normal

### Effect of dietary factors on glucose intolerance

The age-adjusted mean values of baseline energy ratios of major nutrients and energy-adjusted nutrition intake in relation to sex and glucose tolerance status at the end of the follow-up period are depicted in Table 2. In males, subjects with future GI had slightly higher total energy intake and lower energy ratio of carbohydrates than did normal subjects. The amount of complex carbohydrate intake was slightly lower, but that of dietary cholesterol and ethanol intake was higher in the GI male subjects than in normal subjects; the difference in the amount of ethanol intake was statistically significant (26.7 vs. 15.7 g, p<0.05). The GI females had slightly but significantly higher mean values of the polyunsaturated/saturated fatty acids (P/S) ratio (1.42 vs. 1.31, p<0.05). The average amounts of other nutrients were not significantly different between GI and normal subjects of either sex.

**Table 2.** Age-adjusted mean values (95% confidence intervals) of baseline energy ratio of major nutrients and nutritional elements by sex and glucose tolerance status.

Nutrient	Males					Females		
	
	Normal		Glucose Intolerance			Normal	Glucose Intolerance	
	n=352		n=58			n=580	n=85	
Energy (kcal)	1947	(1903, 1990)	2048	(1941, 2155) †	1598	(1574, 1621)	1581	(1519, 1644)
Protein (% energy)	12.7	(12.5, 13.0)	12.4	(11.9, 13.0)	13.5	(13.4, 13.7)	13.7	(13.3, 14.1)
Total fat (% energy)	24.2	(23.6, 24.7)	23.2	(21.8,24.5)	27.9	(27.5, 28.4)	28.3	(27.2, 29.4)
Carbohydrates (% energy)	54.9	(54.1, 55.7)	52.9	(50.9, 54.9) †	55.6	(55.1, 56.1)	55.4	(54.0, 56.7)

Protein (g)	54.9	(53.7, 56.1)	52.8	(49.8, 55.8)	58.6	(57.9, 59.3)	59.4	(57.7, 61.2)
Animal protein (g)	21.2	(20.2, 22.2)	22.0	(19.5, 24.5)	21.6	(21.1-22.2)	21.2	(19.8, 22.6)
Total fat (g)	46.7	(45.5, 48.0)	44.8	(41.8, 47.9)	52.9	(52.1, 53.7)	53.5	(51.4, 55.6)
Animal fat (g)	16.4	(15.5, 17.2)	15.3	(13.2, 17.4)	17.8	(17.2, 18.4)	16.4	(14.7, 18.0)
Polyunsaturated fatty acids (g)	15.2	(14.6, 15.8)	14.4	(12.9, 15.8)	17.7	(17.3, 18.1)	18.5	(17.5, 19.5)
Monounsaturated fatty acids (g)	18.7	(18.2, 19.1)	18.5	(17.4, 19.5)	20.7	(20.3, 21.0)	20.9	(20.1, 21.7)
Saturated fatty acids (g)	12.4	(12.0, 12.8)	11.6	(10.6, 12.6)	14.0	(13.7, 14.3)	13.6	(12.8, 14.3)
P/S ratio	1.31	(1.26, 1.36)	1.36	(1.24, 1.48)	1.31	(1.27, 1.35)	1.42	(1.32, 1.51)**
Carbohydrates (g)	239.6	(235.4, 243.8)	231.0	(220.6, 241.3)	237.0	(234.8, 239.2)	236.0	(229.7, 241.3)
Simple (g)	34.7	(32.2, 37.1)	35.8	(29.8, 41.8)	38.0	(36.5, 39.5)	38.8	(34.8, 42.7)
Complex (g)	204.9	(200.7, 209.2)	195.2	(184.7, 205.6) †	199.0	(196.7, 201.3)	196.8	(190.8, 202.8)
Dietary fiber (g)	3.8	(3.7, 3.9)	3.5	(3.2, 3.9)	4.5	(4.4, 4.6)	4.7	(4.4, 5.0)
Dietary cholesterol (mg)	237.4	(226.1, 248.7)	263.9	(236.0, 291.8) †	254.8	(247.9, 261.7)	251.6	(233.5, 269.7)
Ethanol (g)	15.7	(13.2, 18.2)	26.7	(20.4, 33.0)*	5.4	(4.7, 6.2)	5.1	(3.1, 7.1)

Nutritional elements were adjusted for energy intake using the method of Willet and Stampfer.^22^

Glucose Intolerance: impaired glucose tolerance and diabetes by the 1998 World Health Organization criteria

**: p<0.01, *: p<0.05, † : p<0.1 vs Normal

Table 3 compares the age-adjusted mean values of changes in the energy ratio of major nutrients and energy-adjusted nutrition intake from 1988 to 1993/1994 between the groups. There was no difference in changes in the total energy intake and the energy ratios of major nutrients in either sex. The amount of protein intake increased more in the male subjects who developed GI than in those who did not (albeit to marginally significant, p<0.1). For females, the amount of animal fat less decreased in the GI group than in the normal group (-0.1 g vs. -1.7g), resulting in a significantly decreased P/S ratio in the GI subjects compared with normal subjects (-0.09 vs. 0.05, p<0.05). None of the other nutrients, i.e., carbohydrates, dietary fibers, cholesterol, or ethanol, showed any significant changes.

**Table 3.** Age-adjusted mean values (95% confidence intervals) of changes in energy ratio of major nutrients and amount of nutritional elements from 1988 through 1993/1994 by sex and glucose tolerance status.

Nutrient	Males				Females			
	
	Normal		Glucose Intolerance		Normal		Glucose Intolerance	
	n=352		n=58		n=580		n=85	
Energy (kcal)	-120	(-160, -79)	-129	(-229, -28)	-54	(-79, -29)	-74	(-141, -8)
Protein (% energy)	0.2	(-0.02, 0.5)	0.7	(0.03, 1.3)	0.4	(0.2, 0.5)	0.5	(0.05, 0.9)
Total fat (% energy)	0.1	(-0.5, 0.6)	0.2	(-1.2, 1.6)	-0.04	(-0.5, 0.4)	-0.03	(-1.2, 1.1)
Carbohydrates (% energy)	-0.5	(-1.2, 0.2)	-0.6	(-2.4, 1.2)	-0.4	(-0.9, 0.1)	-0.5	(-1.8, 0.9)

Protein (g)	0.7	(-1.1, 2.5)	5.2	(0.7, 9.6) †	-2.4	(-3.6, -1.3)	-2.9	(-5.8, 0.01)
Animal protein (g)	1.2	(-0.2, 2.5)	2.0	(-1.2, 5.2)	0.1	(-0.5, 0.7)	0.3	(-1.4, 1.9)
Total fat (g)	-0.1	(-1.6, 1.4)	1.6	(-2.1, 5.2)	-3.4	(-4.5, -2.3)	-4.1	(-7.0, -1.2)
Animal fat (g)	-0.4	(-1.3, 0.5)	-1.4	(-3.6, 0.9)	-1.7	(-2.4, 1.1)	-0.1	(-1.8, 1.5) †
Polyunsaturated fatty acids (g)	0.02	(-0.7, 0.8)	1.4	(-0.5, 3.2)	-1.1	(-1.6, -0.6)	-1.7	(-2.4, -1.1)
Monounsaturated fatty acids (g)	-0.04	(-0.6, 0.5)	0.01	(-1.3, 1.3)	-1.2	(-1.6, -0.8)	-2.0	(-3.0, -0.9)
Saturated fatty acids (g)	-0.2	(-0.6, 0.3)	0.2	(-0.9, 1.3)	1.1	(-1.5, 0.8)	-0.4	(-1.3, 0.5)
P/S ratio	0.01	(-0.05, 0.07)	0.05	(-0.1, 0.2)	0.05	(0.01, 0.09)	-0.09	(-0.2, 0.01)*
Carbohydrates (g)	-5.3	(-11.4, 0.8)	-1.7	(-16.7, 13.4)	-17.2	(-20.5, -13.9)	-21.6	(-30.3, 12.9)
Simple (g)	-1.7	(-3.9, 0.6)	-6.3	(-11.9, -0.8)	-2.7	(-4.4, -1.1)	-4.3	(-8.7, 0.06)
Complex (g)	-3.6	(-9.4, 2.2)	4.7	(-9.6, 18.9)	-14.5	(-17.6, -11.4)	-17.3	(-25.4, -9.1)
Dietary fiber (g)	-0.5	(-0.6, -0.3)	-0.07	(-0.5, 0.3)	-0.7	(-0.8, -0.6)	-0.8	(-1.1, -0.5)
Dietary cholesterol (mg)	1.5	(-11.5, 14.5)	-25.7	(-57.8, 6.4)	-16.5	(-24.9, -8.2)	-22.8	(-44.8, -0.9)
Ethanol (g)	0.1	(-1.8, 2.0)	-2.1	(-6.8, 2.6)	-0.5	(-0.8, -0.1)	-0.8	(-1.7, 0.2)

Nutritional elements were adjusted for energy intake using the method of Willet and Stampfer.^22^

Glucose Intolerance: impaired glucose tolerance and diabetes by the 1998 World Health Organization criteria

*: p<0.05, † : p<0.1 vs Normal

### Independent risk factors for glucose intolerance

In order to examine the net effect of risk factors on the development of GI, we performed multiple logistic regression analyses (Table 4) using the marginally significant and significant nutrition intake and non-dietary risk factors such as age, fasting serum insulin, physical activity, body mass index, and smoking habits in each sex. Ethanol intake remained a significant independent risk factor for the development of GI in males (odds ratio for an increase of 10 g, 1.19; 95% confidence interval, 1.08-1.33), while the decreased P/S ratio during the follow-up period was an independent risk factor in females (odds ratio for an increase of 0.1, 0.94; 95% confidence interval, 0.90-0.99). Among non-dietary factors, fasting serum insulin was found to be an independent risk factor for GI in both sexes, and age was an additional risk factor in females.

**Table 4.** Stepwise multiple logistic regression analysis of dietary and non-dietary risk factors for development of glucose intolerance by sex.

Risk Factor	Males		Females	
	
	Odds ratio	95%CI	Odds ratio	95%CI
Ethanol intake (10 g)	1.19	1.08 - 1.33**		
Dietary cholesterol (10 mg)	1.02	0.99 - 1.04 †		
Total energy (1kcal)	NS			
Complex carbohydrates (1g)	NS			
P/S ratio (0.1)			NS	
Change in P/S ratio (0.1)			0.94	0.90 - 0.99*
Change in animal fat (1g)			NS	
Change in protein (1g)	NS			

Age (10 years)	NS		1.68	1.28 - 2.20**
Fasting insulin (1 logarithm)	2.51	1.24 - 4.70**	1.88	1.07 - 3.28*
Physically active on leisure time (yes)	0.50	0.22 - 1.12 †	NS	
Body mass index (1kg/m^2^)	NS		NS	
Smoking (yes)	NS		NS	

Odds ratios represent risk for a difference indicated in parentheses

Blank column: not used in the analysis

CI: confidence interval

NS: not stayed in the final model due to p≥1.0

**: p<0.01, *: p<0.05, †: p<0.1

## DISCUSSION

### Importance of dietary factors

The present study revealed that, among dietary factors, baseline ethanol intake was significantly associated with the development of GI in males, while the decreased P/S ratio during the follow-up was a significant risk factor for GI in females. These associations remained significant even after controlling for other dietary and non-dietary factors such as age, body mass index, serum insulin, smoking and physical activity. Since most of our GI cases consisted of subjects with IGT, these nutrients were considered to be risk factors for the initiation of glucose abnormalities.

The males and females who developed GI had hallmarks of the metabolic syndrome such as concomitant increases in body mass index, blood pressures, triglycerides, serum insulin, and fasting glucose (Table 1). Insulin resistance was a central feature of the metabolic syndrome, and our previous study has identified insulin resistance as the major cause of GI in this population,^25^ which may reflect a genetic predisposition to GI. In our subjects, however, dietary factors such as alcohol consumption and decreased P/S ratio were significant risk factors for the development of GI independent of metabolic syndrome. These findings support the concept that GI develops on the basis of an interaction between hereditary and environmental factors, and offer evidence that dietary intake, one of the most important environment factors, plays a crucial role in the development of GI in contemporary Japanese.

### Dietary lipid and glucose intolerance

In the female subjects in the present study, the intake of animal fat less decreased in the GI group compared with the group of normal subjects, leading to a significant decrease in the P/S ratio in the former group. Several cross-sectional studies have indicated close associations of an increased intake of animal fat and saturated fatty acids with insulin resistance and hyperinsulinemia,^6^^,^^11^ which play a fundamental role in the development of GI. Our data are consistent with these findings and suggest that an increased consumption of animal fat has a major causative effect on the development of GI in Japanese.

Among the females in the present study, the baseline P/S ratio was significantly higher in the GI subjects than in the normal subjects, suggesting that the former might initially have a more healthy diet than did the latter. In addition, none of the dietary factors, with the exception of baseline alcohol consumption, were risk factors for the development of GI in the male subjects. We did not give the participants systematic advice on diet, and the majority of the subjects with later developed GI who were all normal at baseline were considered to not be aware of their own decreased glucose tolerance status until the follow-up OGTT. Since the GI subjects included more persons with obesity, hypertension and hyperlipidemia (Table 1), they may have been more inclined to maintain a healthy diet at baseline and during the follow-up period. In females, however, the dietary pattern was reversed between the two groups during the follow-up period. Although the reason for this sex difference is not evident, one possibility is that females, because they are more likely to prefer to confectionery enriched with animal fat such as dairy product, might be difficult to maintain a healthy diet for a long time.

### Westernization of dietary habits and glucose intolerance

There have been several reports suggesting that the Japanese may have an inherent risk of diabetes,^4^^,^^5^ and also that the westernization- associated changes in environmental factors, especially dietary, may increase this risk. In a study comparing Japanese migrants and their offspring in Hawaii and Japanese living in Hiroshima, Kawate et al.^4^ have indicated that the prevalence of diabetes among Japanese-Americans is 1.8 times higher than that among native Japanese. In this study, no difference in total energy intake was observed between the two groups, but the consumption of animal fat and simple carbohydrates was at least twice as high among Japanese-Americans. In contrast, Japanese in Hiroshima consumed about twice the amount of complex carbohydrates as Japanese in Hawaii. These observations support the hypothesis that a high-fat, high-simple carbohydrate, and low-complex carbohydrate diet increases the risk of diabetes, especially in Japanese. These findings are in accord with the recent trends in dietary style^26^ and the increasing prevalence of diabetes in our country,^2^^,^^18^^,^^27^ implying that the risk of diabetes may increase still further in the future: if the westernization of the Japanese life-style, including dietary intake, also continues to advance.

### Alcohol and glucose intolerance

Among the males in the present study, baseline alcohol consumption was identified as a risk factor for future GI. However, this association could not be found in females, since the majority of female subjects did not consume alcoholic beverages or consumed only small amounts of alcohol. Recently, several cross-sectional epidemiologic studies have indicated the possibility that a small amount of alcohol intake reduces hyperinsulinemia, and thus that low doses of alcohol have a protective effect against insulin resistance and subsequent diabetes.^28^^,^^29^ On the other hand, a dose-response relationship between an alcohol intake of eight drinks per day and postloading blood glucose level has been shown in a cross-sectional survey of a large Kaiser-Permanente cohort in the United State.^30^ Our previous prevalence study also showed a significant association of alcohol intake with diabetes.^25^ Yki-Jävinen et al.^31^ have demonstrated in a clinical study using the euglycemic insulin clamp technique that moderate to high alcohol intake increases insulin resistance and reduces glucose metabolism. It has also been reported that alcohol intake increases the levels of diols in blood, an intermediate of alcohol metabolism, which powerfully suppresses the insulin action on fat tissue.^32^ These previous and our present findings provide evidence that alcohol intake is closely related to the development of GI.

### Limitation

First, it is probable that some heavy drinkers among our subjects underreported their alcohol intake. However, the findings of our study do not seem to be results of this under-reporting, since all subjects were of normal glucose tolerance at baseline, and under-reporting is considered to have occurred randomly in both groups irrespective of future glucose tolerance status. Second, the information regarding nutrient intake was derived from a semi-quantitative food frequency questionnaire that asked about average intake over the previous year. The limitations of this method are well known,^33^ and random measurement error is likely to have contributed a bias toward a finding of no effect. Therefore, the estimates of effect that we have found are probably conservative. Third, our results might be biased by exclusion of subjects who did not return for the follow-up examination. At baseline, male subjects with missing values for follow-up were younger than those who were followed up (53.8 vs. 55.3 years, p=0.001, in males; 54.9 vs. 54.6 years, p=0.74, in females). In females, subjects without follow-up examination had a lower mean value of baseline body mass index (22.4 vs. 22.9 kg/m^2^, p=0.02) and a higher mean value of systolic blood pressure (128 vs. 126 mmHg, p=0.04) than others, but such differences were not observed in males. In regard to dietary factors, baseline energy intake was lower in subjects without follow-up examination than in those with follow-up examination for both sexes (1880 vs. 1965 kcal, p=0.01, in males; 1523 vs. 1595 kcal, p=0.007, in females). Males without follow-up examination had lower intake of carbohydrates compared with those with follow-up examination (228.5 vs. 236.4 g, p=0.01), while females without follow-up examination had lower intake of protein (56.2 vs. 58.0 g, p=0.02). However, these facts make it unlikely that this selection bias invalidates the findings of the present study.

### Conclusion

The results show that, in addition to fasting insulin, nutritional factors also predict the development of GI. A decreased P/S ratio is an independent risk factor for GI in females, as is alcohol intake in males. For the primary prevention of diabetes, of which the occurrence has increased steeply in Japan, it is important to restrict the westernization of dietary habits in addition to decreasing the incidence of both a sedentary lifestyle and obesity.
